# Diagnostic Potential of Differentially Expressed Homer1, IL-1β, and TNF-α in Coronary Artery Disease

**DOI:** 10.3390/ijms16010535

**Published:** 2014-12-29

**Authors:** Xuan Jing, Shan-Shan Chen, Wei Jing, Qian Tan, Ming-Xia Yu, Jian-Cheng Tu

**Affiliations:** Department of Clinical Laboratory Medicine and Center for Gene Diagnosis, Zhongnan Hospital of Wuhan University, Wuhan 430071, China; E-Mails: jx05070103@163.com (X.J.); css19881114@126.com (S.-S.C.); xjw2009@yeah.net (W.J.); silverlingapple@163.com (Q.T.); dewrosy520@163.com (M.-X.Y.)

**Keywords:** coronary artery disease, gene expression, inflammation, Homer, IL-1β, TNF-α

## Abstract

Increasing evidences suggest that inflammation plays an important role in the pathogenesis of coronary artery disease (CAD). Numerous inflammatory cytokines and related genes mediate adverse cardiovascular events in patients with CAD, such as interleukin-1β (IL-1β), tumor necrosis factor-α (TNF-α), and Homer in the present study. The study was carried out on 163 CAD patients at different stages and 68 controls. The gene expression of Homer1, Homer2, Homer3, IL-1β, and TNF-α in the peripheral blood leukocytes were measured by real-time polymerase chain reaction. The mRNA levels of Homer1, IL-1β, and TNF-α in CAD patients were significantly higher than those in the control group, but not Homer2 and Homer3. However, there was no considerable difference in the mRNA levels of Homer1, IL-1β, and TNF-α among AMI, UAP, and SAP three subgroups of CAD. The receiver operating characteristic (ROC) curves showed that Homer1 had a better diagnostic value for UAP patients compared with IL-1β and TNF-α. Like IL-1β and TNF-α, Homer1 may also be an important participant of atherosclerotic plaque development and eventually rupture. The results of the present study may provide an important basis for diagnosing CAD patients, and provide new therapeutic targets for CAD.

## 1. Introduction

Coronary artery disease (CAD) has become a public health problem with high morbidity and mortality worldwide, with an estimated more than 700,000 deaths annually in China [1,2]. Patients with CAD can present with stable angina pectoris (SAP), unstable angina pectoris (UAP), or acute myocardial infarction (AMI). Many studies showed that CAD is mainly caused by an interaction between genetic and environmental factors [3]. In the past decades, many contributing factors including a family history of premature CAD, diabetes mellitus (DM), cigarette smoking, hypertension, hyperlipidemia, atherosclerosis, obesity, and high-fat/low-fiber diets have been established, but the exact pathogenesis of CAD is not fully understood yet. However, increasing evidences suggest that inflammation plays an important role in the pathogenesis of both the chronic and acute phases of CAD [4,5,6]. The common cause of CAD is the formation of plaque and the rupture of the unstable atherosclerotic plaque. Recent molecular studies have shown altered mRNA level of many genes in both atherosclerotic plaque and peripheral blood cells may associate with CAD [7].

CAD is a chronic inflammatory disease. Atherosclerosis, the pathological formation of atherosclerotic plaques in one or more of the coronary arteries, is the leading cause of CAD [8]. Platelet secretion and aggregation as well as thrombus formation of blood platelets are critically associated with increased cytosolic Ca^2+^ concentration, mainly due to the release of intracellular Ca^2+^ followed by store-operated Ca^2+^ entry (SOCE) through Ca^2+^ release-activated Ca^2+^ (CRAC). The major players in the SOCE pathway are a Ca^2+^ sensor protein, STIM1, and a channel subunit, Orai 1 [9]. Previous study have found that both Homer and nuclear factor-kappaB (NF-κB) could upregulate STIM1 and Orai1 thus SOCE [10,11], and then regulate the cytosolic Ca^2+^ concentration.

NF-κB, a critical regulator of innate and adaptive immunity, is critically important for regulating many key inflammatory genes linked to atherosclerosis [12]. NF-κB activation is required for pro-inflammatory response [13]. Pro-inflammatory cytokines, such as IL-1β and TNF-α, play a critical role in contributing to the atherosclerotic process. TNF-α, mainly produced by activated macrophages, participates in the vasodilatation and edema formation, and mediates the recruitment of neutrophils and macrophages to sites of inflammation by stimulating endothelial cells to produce adhesion molecules [14]. IL-1β, also produced by activated macrophages, can promote the activation of T and B cells in addition to the similar function with TNF-α. The pro-inflammatory cytokines ultimately active the transcription factor NF-κB, and then indirectly regulate the cytosolic Ca^2+^ concentration, participating in the platelet activation.

Homer, known as a scaffolding protein, includes three subtypes (Homer1, Homer2, Homer3) and several splice variants [15,16,17,18]. Homer was best defined in the nervous system before, however, more and more researchers began to investigate the role of Homer in cardiovascular disease [19,20]. As a cytosolic adaptor, Homer plays different roles in cell function, including the regulation of G-protein-coupled receptors [21]. Previous studies [10,22] have shown that Homer can up-regulate SOCE, and then, mediate the intracellular Ca^2+^ concentration, thus, Homer may play an important role in platelet aggregation.

The aim of the present study was to investigate the gene expression of Homer1, IL-1β, and TNF-α in the peripheral blood leukocytes from CAD patients, and to provide an important basis for diagnosing CAD patients, and what’s more, to provide a new therapeutic target for CAD.

## 2. Results

### 2.1. Patient Characteristics

The main demographic and clinical characteristics of all the studied subjects were summarized in Table 1. There was no significant difference in important risk factors including age, gender, BMI, smoking, hypertension, diabetes, hypercholesterolemia, CHOL, TG, and LDL-C in the four groups. HDL-C and GLU showed significant differences between AMI and the control group (AMI* vs.* Control: *p* < 0.05). Lp (a) of CAD group (AMI, UAP, and SAP) was much higher than the control group (AMI* vs.* Control: *p* < 0.01; UAP* vs.* Control: *p* < 0.01; SAP* vs.* Control: *p* < 0.05).

**Table 1:** Characteristics of coronary artery disease (CAD) cases and the control group.

Characteristics	Control	AMI	UAP	SAP
	(*n* = 68)	(*n* = 65)	(*n* = 53)	(*n* = 45)
Sex (M/F)	40/28	47/18	38/15	33/12
Age (years)	62.44 ± 0.70	62.80 ± 1.39	63.29 ± 1.12	63.01 ± 0.93
BMI (kg/m^2^)	24.51 ± 3.31	25.90 ± 4.03	26.12 ± 4.17	25.77 ± 3.39
Somking, *n* (%)	12 (17.6%)	10 (15.4%)	7 (13.2%)	7 (15.6%)
Hypertension, *n* (%)	31 (45.6%)	35 (53.8%)	28 (52.8%)	23 (51.1%)
Diabetes, *n* (%)	18 (26.4%)	25 (38.5%)	21 (39.6%)	17 (37.8%)
Hypercholesterolemia	3 (4.4%)	7 (11.5%)	6 (11.3%)	3 (6.7%)
CHOL (mg/dL)	155.45 ± 15.85	171.69 ± 8.89	168.98 ± 8.12	159.70 ± 14.69
TG (mg/dL)	128.39 ± 38.95	166.46 ± 24.79	147.87 ± 30.10	143.44 ± 23.90
HDL-C (mg/dL)	49.49 ± 25.90	37.13 ± 15.46 *	41.77 ± 30.16	43.70 ± 13.14
LDL-C (mg/dL)	103.24 ± 35.18	105.57 ± 33.64	95.13 ± 25.52	101.32 ± 32.09
GLU (mg/dL)	98.75 ± 21.98	112.63 ± 37.84	107.76 ± 24.68	101.99 ± 27.75
Lp (a) (mg/L)	211.20 ± 11.17	278.21 ± 23.01 **	283.70 ± 33.32 **	244.63 ± 13.60 *

Data are mean ± SD or percentage. * *p* < 0.05, ** *p* < 0.01 *vs.* Control. Abbrebiations: CAD: coronary artery disease; BMI: body mass index; CHOL: total cholesterol; TG: triglycerides; HDL-C: High-density lipoprotein cholesterol; LDL-C: Low-density lipoprotein cholesterol; GLU: Fasting glucose; Lp (a): lipoprotein (a).

### 2.2. The mRNA Levels of Homer and the Pro-Inflammatory Cytokine IL-1β and TNF-α in Peripheral Blood Leukocytes from CAD Patients

**Figure 1:**
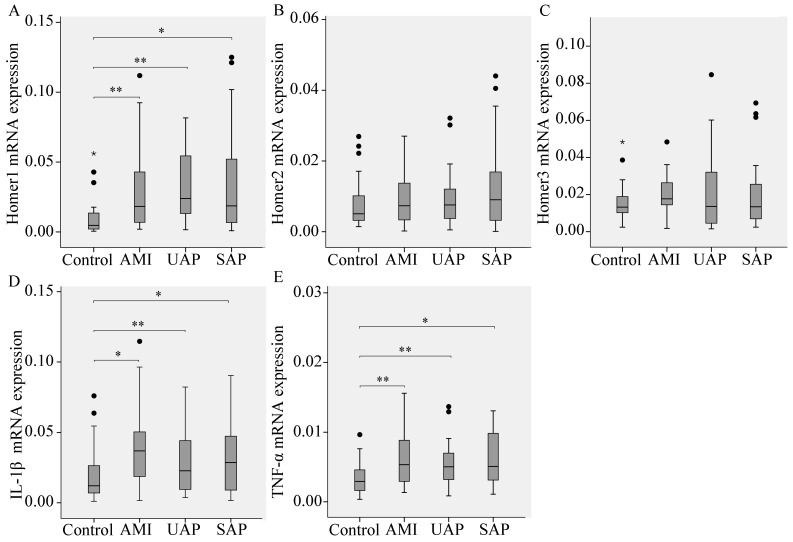
The expression of Homer1 (**A**); Homer2 (**B**); Homer3 (**C**) and the pro-inflammatory cytokine interleukin-1β (IL-1β) (**D**); and tumor necrosis factor-α (TNF-α) (**E**) in peripheral blood leukocytes from CAD patients. Results are shown as median box and whisker plots. Statistical differences in relative expression between CAD patients and controls are indicated as asterisks: * (*p* value, Mann-Whitney test). * *p* < 0.05, ** *p* < 0.01 *vs.* Control. “•” represents outliers; “*” represents extreme values.

Expression of Homer1, Homer2, Homer3, and the pro-inflammatory cytokines IL-1β and TNF-α relative to GAPDH in CAD patients compared with control subjects were performed. Results showed that the mRNA levels of Homer1, IL-1β, and TNF-α in the AMI, UAP and SAP subgroups were all higher than those in the control group, however, there was no significant difference in the mRNA levels of Homer2 and Homer3 between CAD and the control group, suggesting that Homer1, IL-1β, and TNF-α were related to CAD pathogenesis. Comparing the mRNA levels of three subgroups of CAD subjects, no remarkable differences were found among these groups (Figure 1).

### 2.3. Subgroup Analyses

The gene expression of Homer1, Homer2, Homer3, IL-1β, and TNF-α were analyzed by hypertension and diabetes between CAD patients and controls (Table 2). For Homer1, the gene expression differed significantly between CAD patients and controls in participants of hypertension (*p* < 0.001), normal blood pressure (*p* = 0.001), diabetes (*p* = 0.01), and normal glucose (*p* < 0.001). For IL-1β and TNF-α, there were significant differences between CAD patients and controls in participants of hypertension (*p* < 0.001 and *p* < 0.001), diabetes (*p* = 0.017 and *p* = 0.025), respectively.

**Table 2:** Subgroup analyses of the gene expression of Homer1, Homer2, Homer3, IL-1β, and TNF-α between CAD patients and controls by hypertension and diabetes.

Gene	Subgroup	Sample Size		Expression *		*p*
		Controls	CAD	Controls	CAD	
Homer1	Hypertension	31	86	0.012 (0.007, 0.018)	0.031 (0.018, 0.082)	<0.001
	Normotension	37	77	0.003 (0.002, 0.008)	0.009 (0.004, 0.022)	0.001
	Diabetes	18	63	0.016 (0.010, 0.022)	0.028 (0.013, 0.080)	0.001
	Euglycemia	50	100	0.003 (0.001, 0.008)	0.017 (0.007, 0.044)	<0.001
Homer2	Hypertension	31	86	0.007 (0.003, 0.012)	0.008 (0.004, 0.012)	0.602
	Normotension	37	77	0.006 (0.004, 0.009)	0.007 (0.003, 0.014)	0.635
	Diabetes	18	63	0.005 (0.003, 0.013)	0.008 (0.003, 0.011)	0.479
	Euglycemia	50	100	0.006 (0.004, 0.009)	0.007 (0.003, 0.017)	0.473
Homer3	Hypertension	31	86	0.011 (0.008, 0.014)	0.012 (0.006, 0.025)	0.693
	Normotension	37	77	0.015 (0.010, 0.026)	0.011 (0.005, 0.019)	0.065
	Diabetes	18	63	0.015 (0.013, 0.027)	0.014 (0.005, 0.028)	0.428
	Euglycemia	50	100	0.011 (0.008, 0.017)	0.009 (0.005, 0.019)	0.316
IL-1β	Hypertension	31	86	0.014 (0.007, 0.024)	0.035 (0.019, 0.058)	<0.001
	Normotension	37	77	0.011 (0.007, 0.032)	0.021 (0.007, 0.029)	0.162
	Diabetes	18	63	0.019 (0.009, 0.034)	0.031 (0.016, 0.051)	0.017
	Euglycemia	50	100	0.011 (0.007, 0.023)	0.018 (0.009, 0.026)	0.241
TNF-α	Hypertension	31	86	0.003 (0.002, 0.004)	0.006 (0.005, 0.010)	<0.001
	Normotension	37	77	0.002 (0.001, 0.006)	0.003 (0.002, 0.006)	0.148
	Diabetes	18	63	0.006 (0.003, 0.008)	0.008 (0.006, 0.013)	0.025
	Euglycemia	50	100	0.002 (0.001, 0.005)	0.003 (0.002, 0.006)	0.121

Abbreviation: CAD: coronary artery disease; IL-1β: interleukin-1β; TNF-α: tumor necrosis factor-α. * Expression: Median (25 Percentiles, 75 Percentiles).

### 2.4. The Diagnostic Value of Homer1, IL-1β, and TNF-α in AMI, UAP, and SAP Respectively

**Figure 2:**
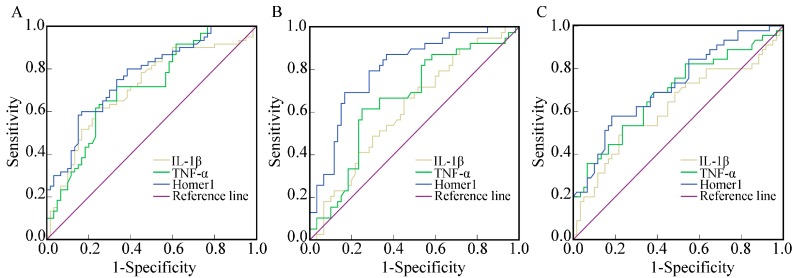
Receiver operating characteristic (ROC) curves. The ROC curves of the expression of Homer1, IL-1β, and TNF-α for acute myocardial infarction (AMI) (**A**); unstable angina pectoris (UAP) (**B**); and stable angina pectoris (SAP) (**C**) groups.

The area under the receiver operating characteristic curves (AUC_ROC_) indicated that Homer1 together with IL-1β and TNF-α could be thought as potent biomarkers for CAD (Figure 2 and Table 3). The data also showed that Homer1 could have a better diagnostic value for UAP patients compared with IL-1β and TNF-α, however, the differences of diagnostic value among Homer1, IL-1β, and TNF-α for AMI and SAP groups were not obvious.

**Table 3:** Comparisons of the AUC (95% CI) of the expression of Homer1, IL-1β, and TNF-α for AMI, UAP, and SAP groups.

Group	Gene	AUC	95% CI	*p*	Se (%)	Sp (%)
AMI	IL-1β	0.711	0.617–0.805	<0.0001	61.7	76.7
	TNF	0.713	0.612–0.804	<0.0001	61.7	76.7
	Homer1	0.766	0.682–0.849	<0.0001	58.3	85.0
UAP	IL-1β	0.618	0.506–0.729	0.049	66.7	55.0
	TNF	0.659	0.548–0.769	0.008	61.5	75.0
	Homer1	0.803	0.715–0.890	<0.0001	69.2	83.3
SAP	IL-1β	0.620	0.507–0.732	0.036	53.3	76.7
	TNF	0.687	0.583–0.792	0.001	68.9	61.7
	Homer1	0.720	0.622–0.818	<0.0001	57.8	81.7

Abbreviation: AUC: area under the receiver operating characteristic curves; Se: sensitivity; Sp: specificity; AMI: acute myocardial infarction; UAP: unstable angina pectoris; SAP: stable angina pectoris.

## 3. Discussion

CAD is a chronic human disease with leading cause of premature morbidity and mortality throughout the entire world. Inflammation plays an important role in the pathogenesis of CAD. There is ample evidence for the role of inflammation in all stages of the atherosclerotic disease process [23,24,25]. In the present study, we investigated the gene expression of Homer1, Homer2, Homer3, and pro-inflammatory cytokines, IL-1β and TNF-α in the peripheral blood leukocytes, which may all relate to the inflammatory immune response. In our study, we demonstrated that the gene expression of Homer1, IL-1β and TNF-α in the peripheral blood leukocytes were associated with CAD, but not Homer2 and Homer3. The subgroup analyses showed that hypertension and diabetes may have some relevance with the gene expression of IL-1β and TNF-α. The area under the AUC_ROC_ showed that Homer1 had a better diagnostic value for UAP patients compared with IL-1β and TNF-α, and the differences of diagnostic value for AMI and SAP groups among Homer1, IL-1β, and TNF-α were not obvious. However, the limitations of the study are that the sample size is small and the exact meaning of this phenomenon remains to be further demonstrated.

As we all known, atherosclerosis, characterized by the formation of atherosclerotic plaques, is the underlying cause of CAD. It is a chronic immune-inflammatory condition in which the interactions of monocytes with activated endothelium are crucial events leading to atherosclerotic alteration of the arterial intima [26]. In early atherosclerosis, the local endothelial-cell defect promotes the adhesion of leukocytes and migration of activated platelets to the damaged endothelium and leads to an increased permeability of blood vessels for plasma lipid components [27].

Platelet’s activation is critically associated with increased cytosolic Ca^2+^ concentration. Homer, in the present study, as a cytosolic adaptor, could regulate the concentration of cytosolic Ca^2+^. Jardin *et al*. [10] showed that Homer played an important role in thrombin-stimulated platelet function, which was likely to be mediated by the support of agonist-induced Ca^2+^ entry. Many evidences prove that SOCE in platelets is further regulated by Homer, and therefore Homer plays an important role in agonist-induced platelet aggregation [10,22]. Since Homer’s important role in platelet’s activation and the gene expression of Homer1 has some relevance with CAD in the present study, we may suppose that Homer1 is an important participant in early atherosclerosis, consequently, CAD.

Leukocyte recruitment and expression of pro-inflammatory cytokines characterize early atherogenesis. Monocyte-macrophages constitute the majority of immune cells in the atherosclerotic lesion [28]. Monocyte-macrophage lineage cells are key factors in lesion development, participating in the processes that mediate the progression of the atherosclerotic plaque [29]. Activated macrophages can secrete pro-inflammatory cytokines, including IL-1β and TNF-α, which may influence the mechanisms involved in vessel occlusion and repair [30]. Clinical studies have shown increased expression of IL-1β and TNF-α in the atherosclerotic plaques [31,32,33]. Similarly to these reports, in the current study, we observed increased mRNA levels of IL-1β and TNF-α in the leukocytes of the CAD patients than in the controls. These results have indicated the important role of IL-1β and TNF-α in the progression of CAD.

Recent studies have found that serum concentrations of eotaxin have a distinct relationship with TNF-α, IL-1β, and IL-6 [34,35]. Eotaxin, an eosinophil chemoattractant cytokine, plays an important role in the development of atherosclerosis [36]. In the serum of patients with CAD, increased circulating levels of eotaxin are detected [37]. What’s more interesting is that circulating eotaxin levels combine hepatic steatosis could well predict carotid intima-media thickness (cIMT) in obese patients with nonalcoholic fatty liver disease, a distinct CAD risk factor [34]. IMT, defined as the distance between the lumen-intima and the media-adventitia ultrasound interfaces, is a completely non-invasive and sensitive method to detect early atherosclerosis [38]. A clinical study from Ciccone *et al.* [39] have found levels of inflammatory markers, such as TNF-α and IL-6, are correlated to cIMT in Obstructive Sleep Apnea (OSA), which is a sleep-related breathing disorder associated with the development of cardiovascular diseases and atherosclerosis. In our study, Homer1, like TNF-α and IL-1β, could be a potential and important participant in diagnosing early atherosclerosis, consequently, CAD. While whether there is a correlation between cIMT and Homer1, or whether the combination of cIMT and Homer1 could be a more useful diagnostic method for early atherosclerosis still need further studies.

In the present study, we found that the mRNA levels of Homer1 and pro-inflammatory cytokines, IL-1β and TNF-α in the peripheral blood leukocytes from CAD patients were all increased in various degrees. Maybe they have a synergistic effect on the pathogenesis of CAD through the SOCE mechanism regulating platelet secretion and aggregation as well as the formation of atherosclerotic plaques, and play important roles in the CAD process.

## 4. Experimental Section

### 4.1. Patients

We recruited 163 patients who were diagnosed as CAD by coronary angiography between June 2013 and June 2014 in the Zhongnan Hospital of Wuhan University, Wuhan, China. The CAD diagnosis was based upon stenosis affecting ≥50% of the luminal diameter. Patients were classified into three groups: SAP (33 men and 12 women, mean age 63.01 ± 0.93), UAP (38 men and 15 women, mean age 63.29 ± 1.12), and AMI (47 men and 18 women, mean age 62.8 ± 1.39). Patients with valvular heart disease, thromboembolism, collagen disease, disseminated intravascular coagulation, advanced liver disease, renal failure, malignant disease, or septicemia or that were on steroid therapy were excluded from the study. We also selected 68 subjects as controls, who were undergoing routine medical examinations at the Physical Examination Center. The exclusion criteria included acute or chronic infections or inflammatory diseases; severe pulmonary, hepatic or hematological diseases; acute or chronic renal dialysis; malignant tumors. Those who had a family history of CAD, any clinical manifestations or a medical history of heart disorders, previous percutaneous or surgical myocardial revascularization or abnormal ECG were also excluded.

Written informed consent was obtained from the population involved in this study and the study protocol was approved by the Ethics Committee of Zhongnan Hospital of Wuhan University (Ethic approval 2013059, April 2013).

### 4.2. RNA Isolation

Blood samples were obtained from patients within 24 h after the onset of chest pain. Total mRNA was extracted from peripheral blood leukocytes using the Trizol reagent (Invitrogen, Carlsbad, CA, USA). All the RNA extracts were treated with Dnase I to avoid contamination by genomic DNA. The concentration of the RNA measured by Nanodrop 2000 spectrophotometer (Thermo Scientific Inc., Waltham, MA, USA). cDNA was synthesized using the Maxima First Strand cDNA Synthesis Kit (Thermo Scientific Inc., Waltham, MA, USA). Reverse transcription conditions were as follows: 65 °C for 5 min, and then 42 °C for 60 min, and 70 °C for 5 min.

### 4.3. Real-Time Polymerase Chain Reation

To determine the expression of Homer1, Homer2, Homer3, IL-1β, and TNF-α, relative quantitative real-time polymerase chain reaction (RT-PCR) was performed using SYBR-green I Premix EXTaq on the Bio-Rad CFX96 (Bio-Rad Laboratories, Inc., Hercules, CA, USA) following manufacturer’s instructions. Primers sequences for amplification of Homer1, Homer2, Homer3, IL-1β, and TNF-α were determined using primer 3.0 (Primer Biosoft, Palo Alto, CA, USA) or previously designed [40,41]. The synthesized primers were as follows: Homer1 sense: 5'-GATCCTGCCTAGCCTTCT-3' and antisense: 5'-GGAGCGAGCAACCAAACG-3'; Homer2 sense: 5'-TCACCGTTTCCTACTTCTATG-3' and antisense: 5'-CCTGCGTCTTGTCTT-TGG-3'; Homer3 sense: 5'-CGCACTCACTGTCTCCTATT-3' and antisense: 5'-GGAACTTCTCGG-CAAACT-3'; IL-1β sense: 5'-CCTGTCCTGCGTGTTGAAAGA-3' and antisense: 5'-GGGAACTGGGCAGACTCAAA-3'; TNF-α sense: 5'-AGCCCATGTTGTAGCAAACC-3' and antisense: 5'-TGAGGTACAGGCCCTCTGAT-3'. The Glyceraldehyde-3-phosphate dehydrogenase (GAPDH) was used as the endogenous control and amplified simultaneously with assessed genes. Primers used for GAPDH amplification were as follows: sense: 5'-GAAGGTGAAGGTCGGAGTC-3' and antisense: 5'-GAAGATGGTGATGGGATTTC-3'.

Real-time PCR was carried out in 20 μL of reaction solution (10 μL of SYBR green mix, 0.8 μL 10 μmol sense, 0.8 μL 10 μmol antisense, and 7.4 μL water and 1 μL cDNA). The reactions started at 95 °C for 5 min, followed by 40 cycles of 95 °C for 30 s, 58 °C for 30 s and 72 °C for 30 s. All experiments were carried out in duplicate for each data point. Relative gene expression level (the amount of target, normalized to endogenous control gene) was calculated using the comparative *C*_t_ method formula 2^−ΔΔ*C*t^.

### 4.4. Statistical Analysis

All data were analyzed by SPSS 17.0 (SPSS, Inc. Chicago, IL, USA). Normally distributed data were expressed as mean ± standard deviation. Skewed data were described by the median and interquartile range. Differences were considered to be significant at *p* < 0.05. To check the normality of the distribution, the Shapiro-Wilk test was carried out. The differences between normally distributed numeric variables were evaluated by Student’s *t*-test. Oneway ANOVA was used for the comparison among multi-groups if the variance was homogeneous, while non-normally distributed variables were analyzed by Mann-Whitney U test or Kruskal-Wallis variance analysis, as appropriate. Chi-square test was employed for the comparison of categorical variables. The subgroup analyses of the gene expression of Homer1, Homer2, Homer3, IL-1β, and TNF-α were performed by hypertension and diabetes between CAD patients and controls. The diagnostic performance of the mRNA levels of Homer1, IL-1β, and TNF-α in peripheral blood leukocytes for diagnosis of different stages of CAD patients were examined by the area under the corresponding ROC curve analysis.

## 5. Conclusions

In summary, we have demonstrated that the mRNA levels of Homer1, IL-1β, and TNF-α in peripheral blood leukocytes are closely associated with CAD in the present study. The underlying mechanism may be their regulatory effects on the formation of atherosclerotic plaque, consequently, CAD. This method will provide us a useful auxiliary diagnostic method to screen for a large number of candidate patients and increase our understanding of the processes underlying the pathology of CAD, and then provide new targets for the treatment of inflammatory disease.
